# Exploration of ethno-medicinal knowledge among rural communities of Pearl Valley; Rawalakot, District Poonch Azad Jammu and Kashmir

**DOI:** 10.1371/journal.pone.0183956

**Published:** 2017-09-08

**Authors:** Humaira Shaheen, Mirza Faisal Qaseem, Muhammad Shoaib Amjad, Piero Bruschi

**Affiliations:** 1 Department of Biosciences, COMSATS Institute of Information Technology, Islamabad, Pakistan; 2 Department of Botany, PMAS-University of Arid Agriculture, Rawalpindi, Pakistan; 3 Department of Botany, Women University of Azad Jammu & Kashmir, Bagh, Pakistan; 4 Dept. of AgriFood Production and Environmental Sciences -Laboratories of Applied and Environmental Botany, University of Florence, Florence, Italy; Tallinn University of Technology, ESTONIA

## Abstract

**Background:**

Medicinal plants are the basic source of health care in the Pearl Valley District Poonch, Azad Jammu, and Kashmir. The basic aim of present study was to record information about the use of plants in herbal preparation and quantification of recorded data.

**Materials and methods:**

The research was conducted with the null hypothesis that there was no differential distribution of knowledge among the communities between genders and among different age groups in the study area and across cultural medicinal uses of the plants are similar. To check these hypotheses information about medicinal plants was collected from 46 laypeople and 18 herbalists by using an open ended and semistructured questionnaire. Different ethnobotanical indices were calculated in order to quantify the knowledge on the medicinal plants reported in the study.

**Results:**

Our study recorded 136 species of medicinal plants belonging to 45 families with Asteraceae (14 species) as the dominant family of the area. Decoction (26 species), juice and powder (24 species each) were most common methods of preparation. Spearman’s correlation analysis showed that age and gender had the significant effect on both numbers of mentioned species and different uses. A number of known medicinal plants and the number of different uses (H: 38.51; p < 0.001) differ significantly as indicated by Kruskal-Wallis tests. These results showed that the knowledge about the plant varies among different age groups, which were the first hypothesis of the present study. The highest use values (UVs) were reported for *Berberis lyceum* and *Ajuga bracteosa* (1.13 each) followed by *Abies pindrow* (1.03). Highest informant consensus factor (ICF) values were recorded for digestive system diseases (ICF = 0.90) and muscular and skeletal system diseases (ICF = 0.89). The value of Jaccarad index ranged from 6.11 to 32.97 with an average value of 19.84, percentage of similarity was highest between study area and Pir Lasura National Park (34.62%).

**Conclusion:**

High similarity might be due to the fact that the communities living in these areas have same sociocultural values and have more opportunities to exchange their traditional knowledge. The present study provides practical evidence about the use of medicinal plants among the inhabitants of the Pearl Valley.

## Introduction

Ethnobiological knowledge consists of the body of knowledge, beliefs, traditions, and practices that reflect the perception of the local environment by indigenous communities. Within the field of ethnobiology, several pieces of research have been devoted to the study of plants used for medical purposes, one of the oldest forms of ethnobotanical and ethnomedical research known [1–3].

Quantitative ethnobotany may be defined as "the application of quantitative techniques to the direct analysis of contemporary plant use data" [4, 5]. Quantification and associated hypothesis-testing help to generate quality information, which in turn contributes substantially to resource conservation and development. Further, the application of quantitative techniques to data analysis necessitates refinement of methodologies for data collection. Close attention to methodological issues not only improves the discipline of ethnobotany but also enhances the image of ethnobotany among other scientists " [4, 5]. This paper attempts to highlight these unexpected patterns of ethnobotanical knowledge across age, gender and method used.

The variables known to affect medicinal plant knowledge include education, occupation, age, gender and psychosocial variables [6–10]. Age and gender are generally the factors most examined for their influence on knowledge about plants. One of the most studied resources is medicinal plant knowledge because it is a structural component of local medical systems [11]; it is the focus of this study. Much of this knowledge is traditional, that is, learned long ago and passed on with varying degrees of faithfulness for at least two or three generations. However, ethnobiological knowledge can change rapidly. Every tradition had a beginning cf. [12], and was itself a new creation in its time. Ecosystems change, new plants and animals arrive, and people learn new ways of thinking; ethnobiological systems change accordingly, and are typically flexible and dynamic. Field-workers have observed new knowledge being incorporated into systems around the world.

Divergences in knowledge and practice between two cultural groups that live within the same ecosystem are intriguing as they can provide insight into how the lens of culture can not only alter human viewpoints of the environment but even guide human interactions with resources embedded in the ecosystem. To explore the question of what role culture plays in shaping the human–nature interface, we conducted field research in Pearl Vellay in Rawalakot that hosts an incredibly rich repertoire of cultural, linguistic and biological diversity. We hypothesize that two distinct cultural groups living in the same ecosystem will share a similar pattern of use of wild flora for daily subsistence and medical practices, and that distinctions will arise only for those taxa that play a key role in culture-specific ritual, food or health practices [13].

Researchers have previously studied the association between ethnobotanical knowledge and socio-economic factors. Among the factors previously studied researchers have focused on the age [4, 6, 14, 15], sex, the educational level, origin, and the occupation and the wealth of informantion of informents. Among those, researchers have found that those having a stronger influence on shaping ethnobotanical knowledge distribution are age, sex, education level and wealth.

For example, several studies have found a positive association between age and traditional ethnobotanical knowledge [14], although some other studies have not found such association [6]. In contrast, the differences in ethnobotanical knowledge between men and women seem to be more consistent, with studies finding that men have a larger knowledge than women [6, 14, 16, 17], although the trend seems to be inverse in relation to medicinal plants [18]. Such differences are generally explained by sexual distribution of work [19]. Some research also suggest that ethnobotanical knowledge decreases with the increase of education [15, 20–22] and wealth. Several of those characteristics are also linked to the process of acculturation and the loss of indigenous languages (among indigenous communities) [15, 20, 22]. Some studies highlight the importance of occupation on traditional knowledge [21, 23]. Martínez-Ballesté et al. [21] find that larger involvement in agricultural activities resulted in a loss of traditional ecological knowledge, as a consequence of the environmental transformation and loss of biodiversity. In contrast, those activities more dependent on the natural environment are associated to maintenance of traditional knowledge.

Given those previous findings, we hypothesize that the distribution of traditional knowledge will be patterned across socio-economic characteristics. Specifically, we expect to find that men, older people, people born in the area, and poorer people will have higher levels of traditional knowledge than people without those characteristics. We also hypothesize that people whose occupation depends on the environment, like people who practice extensive agriculture and stockbreeding, might also have larger levels of traditional knowledge.

Ethnobotanical knowledge in Poonch Valley (an administrative division of Azad Jammu and Kashmir) has been scarcely investigated, with the exception of a few studies [24–27] Local people have developed a rich ethnobotanical knowledge and use medicinal plants for treating several common diseases. In particular, traditional healers or herbalists play an important role in ensuring some health service to 75% of the rural population [28]. The study was aimed to record and discuss knowledge on medicinal plant uses in the local traditional practices, including (i) Description of preparation and use of plants as medicines. (ii) The differential distribution of knowledge about plants and medinal properties among sectors of the society, and (iii) identifying new forms of use compared with those reported for other neighbouring areas.

## Materials and methods

### Study area

**Fig 1:**
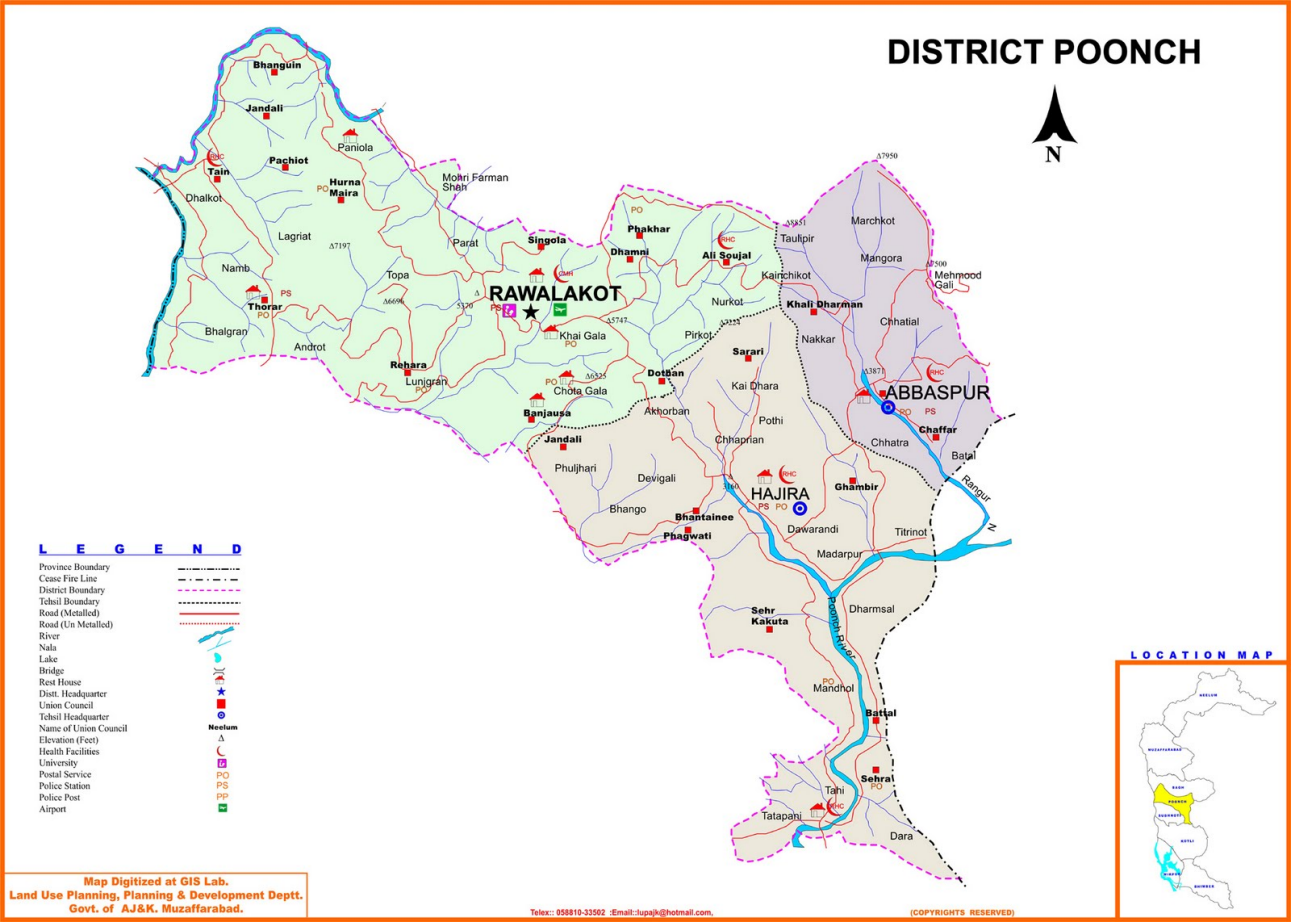
Map of the study area (http://www.pakimag.com/politics/by-election-la-22-sidhnoti-poonch-ajk-assembly.html/attachment/district-poonch-azad-kashmir-detail-map).

The study has been carried out in Rawalakot, also known as Pearl Valley, located in the core of Poonch district (Latitude 33°51'32.18"N, Longitude 73° 45'34.93"E) “Fig 1”. It is a saucer-shaped valley with an altitude of 1615 m a.s.l. The climate can be classified as subtropical highland (Cwa) under the Köppen climate classification due to high altitude. The average annual temperature is 15.3°C ranging from 38°C during the mid-summer months to– 3°C during the winter months. The annual rainfall is very variable year by year and ranges from 500 to 2000 mm, most of which is irregular and falls as intense storms during the monsoon season stretching from July to September. Woodlands, dominated by conifers such as Abies pindrow, Cedrus deodara, Pinus roxburghii, and Pinus wallichiana, and open grasslands, mainly cover the area.

The ethnic composition of the region is quite diverse and complex: Gujjars, Sudhans, Rajputs and Jats are considered to be the major ethnic groups living in the area. Gujjars are the largest group; Rajputs who are spread across the region and Sudhans, mostly settled in Rawalakot, are regarded as the influential ethnic groups in Azad Kashmir. Almost all of them are Muslims. According to the last census in 2014 there are 4,980 households in Rawalakot and each household comprises an average of 7.6 members. This high demographic density can be explained by the higher work opportunities in Rawalakot where most dwellers are engaged, directly or indirectly, with the tourism sector. In contrast, the rural population largely depends on subsidence agriculture, livestock, forestry and formal employment. Agriculture is based on rain-fed cropping system and the main crop of the region is maize.

### Ethnobotanical data collection

Fieldwork was carried out from August 2013 to July 2014. Before initiating of our survey Ethical approval for the study was obtained from the COMSATS Institute of Information Technology ethics Committee. Legal permission was taken from representatives of the municipality for conducting the interview. All respondents were asked to sign a prior informed- consent form after the objectives and possible consequences of the study had been explained. The prior informed consent (PIC) form was translated into the local Pothwari language. Ethnobotanical information was collected from native inhabitants of the valley by using semi-structured questionnaires. This method allows a large number of participants to be interviewed in a relatively short period of time by asking the same questions within a flexible framework. All the interviews were carried out in the local dialect, Pothohari. A total of 64 informants, ranging in age from 35 to 70 years, were selected by convenience sampling (i.e., a sampling method in which units are selected based on easy access or availability) “Table 1”.

**Table 1:** Medicinal Flora of Pearl Valley Tehsil Rawalakot, District Poonch AJ&K.

	Scientific Name and voucher number	Local name and habit	Family	Part Used	Method of preparation/property	Mode of application	Disease treated	FC	RFC	UV
1	*Abies pindrow* Royle (HF-99)	Partal, Paluder silver fir, Tree	Pinaceae	Leaf	paste	External	Swelling	48	0.75	1.03
					Juice	Internal	Antypiretic			
				Bark	Powder	Internal	Cough chronic asthma.			
				Bark	Tea	Internal	Rheumatism			
				Resin	Resin	External	Wounds.			
				Root	Decoction	Internal	Cough, bronchitis			
2	*Achillea millefolium* L. (HF-77)	Yarrow, Herb	Asteraceae	Flower	Extract	Internal	Refrigerante	24	0.38	0.33
				Leaves	Powder	External	Toothache			

3	*Achyranthes aspera* var. *perphyristachya* Hook. F. (HF-128)	Puth kanda, Herb	Amaranthaceae	Root	Decoction	Internal	Inflammation	21	0.33	0.38
				Leaves			Pain			
4	*Adhatoda zeylanica* L. (HF-139)	Bahkar, Herb	Acanthaceae	Bark	Powder	Internal	Stomachache	35	0.55	0.59
							Constipation			
				Leaves			Asthma			
				Root			Cough			
5	*Adiantum capillus-veneris* L. (HF-101)	Hansraj, Sraj fern	Adiantaceae	Leaves	Decoction	Internal	Cough	57	0.89	0.97
							Boils			
							Asthma			
							Jaundice			
							Fever			
							Diabetes			
							Eczema			
							Measles			
							Chest pain			
6	*Adiantum incisum* Foressk (HF-120)	Sumbul, Hansraj, Fern		Leaves	Juice	Internal	Scabies	44	0.69	0.64
							Cough			
							Antypiretic			
							Bodyache			
7	*Aesculus indica* (Wall.ex Camb.) Hook.f. (HF-91)	Bankhore, Horsechestnut, Tree	Hippocastanaceae	Bark	Infusion	Internal	Tonic	33	0.52	0.5
				Fruits	Eaten	Internal	Colic, Rheumatim			
				Seed	Powder	Internal	Leucorrhoea			
8	*Ajuga bracteosa* Wall, ex Benth (HF-82)	Ratti booti, Herb	Lamiaceae	Aerial parts	Extract	Internal	Blood purifier	58	0.91	1
							Pimples			
							Eruption			

				Leaves	Extract	Internal	Inflammation			
							Earache			
							Pain			
							Toothache			
							Boils			
9	*Albizia lebbeck* L. Bth. (HF-89)	Shirin, Tree	Mimoaceae	Seeds		External	Inflammation	57	0.89	0.83
							Leukoderma			
							Leprosy			
							Eczema			
				Bark	Powdered	External	Strengthen spongy gums			
				Bark and seeds	Extract	Internal	Haemorroids			
							Dysentery			
							Diarrhea			
				Flowers	Paste	External	Carbuncles			
							Eczema			
							Swelling			
							Boils			
				Seed	Oil	External	Snake bite			
							Asthma			
10	*Allium griffithianum* Boiss (HF-83)	Piazi, Herb	Alliaceae	Aerial parts	Cooked	Internal	Carminative	29	0.45	0.53
							Dyspepsia			
							Pain			
11	*Alysicarpus bupleurifolius* L. D.C (HF-84)	Buffalo clover, Herb	Paplionaceae	Leaves	Juice	Internal	Blood purifier	15	0.23	0.22
12	*Anaphalis adnata* D.C (HF-20)	Dialect, Herb	Asteraceae	Leaves	Powder	External	Bleeding	19	0.3	0.42
							Wound			
13	*Androsace rotundifolia* Hardwicke (HF-10)	Thandi jari, Herb	Primulaceae	Rhizome	Extract	Internal	Ophthalmic	25	0.39	0.67
				Leaves	Infusion	Internal	Stomachache			
							Emetic			
14	*Anemone tetrasepala* Royle (HF-61)	Herb	Ranunculaceae	Roots	Juice	External	Boils	12	0.19	0.34
15	*Aquilegia pubiflora* Wall ex Royle. (HF-61)	Herb	Ranunculaceae	Root	Paste	External	Snake bite	37	0.58	0.45
							Toothache			
							Emetic			
				Flower	Paste	External	Skin burns			
							Wound			
16	*Artemisia absinthium* L. (HF-46)	Afsanthene, Herb	Asteraceae	Leaves	Infusion, paste	Internal	Anthelmintic	51	0.8	0.98
							Cuts			
							Wounds			
							Stomach disorders			
							Pain			
17	*Artemisia dubia* Wall. ex Bess. (HF-17)	Asfanthene, Herb	Asteraceae	Seeds	Cooked	Internal	Weakness after delivery.	23	0.36	0.52
				Leaves	Paste	External	Cuts			
							Ear diseases			
							Wounds			
				Aerial parts	External	External	Vermicide			
18	*Artemisia maritime* L. (HF-16)	Afsanthene, Herb	Asteraceae	Leaves	Paste	External	Skin infections.	41	0.64	0.77
				Leaf and stem	Powder	Internal	Intestinal parasites.			
19	*Asparagus filicinus* Ham. in D.Don (HF-23)	Macher ghas, Herb	Liliaceae	Root	Decoction	Internal	Nervous stimulant.	38	0.59	0.66
20	*Athyrium tenuifrons* Wall.apud Moore ex. R.Sim (HF-123)	Dandasa, Fern	Adiantaceae	Root	Tea	Internal	Bodyache	32	0.5	0.58
				Root	Powder	External	Wound			
21	*Berberis aristata* DC. (HF-26)	Doody, Indian barberry, Herb	Berberidaceae	Bark	Tooth stick	External	Mouth Inflammation	21	0.33	0.38
				Leaves	Decoction	Internal	Backache			
				Fruit	Paste	External	Dental, Prophylaxis			
22	*Berberis lycium* Royle (HF-81)	Sumblu, Shrub	Berberidaceae	Roots	Extract	Internal	Tonic	59	0.92	0.98
							Pustules			
							Hemorrhoids			
							Diabetes			
							Eye lotion			
				Roots	Paste	External	Skin disease			
							Chronic diarrhea			
							Blood purifier			
							Scabies			
							Fracture			
23	*Bergenia ciliata* (Haw.)Sternb. (HF-18)	Zakhm-e-Hayat, Herb	Saxifragaceae	Aerial parts	Powder	Internal	Urinary tract troubles	29	0.45	0.39
							Earache			
				Leaves	Juice	External	Cough			
				Root	Juice	Internal	Cold			
							Kidney stones			
24	*Calendula officinalis* L. (HF-138)	Sadberga, Herb	Asteraceae	Young branches	Extract	Internal	Kidney stones	46	0.72	0.98
25	*Calotropis procera* (Aiton) W.T.Aiton (HF-59)	Ak, Shurb	Asclepiadaceae	Latex	Latex	External	Aphrodisiacs	35	0.55	0.59
				Root	Power	Internal	Boils			
26	*Caltha alba* var. alba Camb. var. alba (HF-26)	Herb	Ranunculaceae	Aerial parts	Juice	Internal	Antispasmodic	29	0.45	0.28
							Sedative			
27	*Campanula benthamii* Wall. (HF-63)	Herb	Companulaceae	Root	Chewing	External	Strengthen heart	19	0.3	0.36
							Earach			
28	*Carissa opaca* Stapf. ex Haines (HF-32)	Garanda, Small tree	Apocynaceae	Fruit	Powder	Internal	Pain	35	0.55	0.59
				Stem			Inflammation			
29	*Carthamus tinctorius* L. (HF-42)	Kasumba (zafran)	Astreaceae	Flowers	Rubbing	Internal	Pneumonia	29	0.45	0.66
					Boil	External				
30	*Castanea sativa* Mill. (HF-19)	Chita sanghara, Chest nut, Tree	Fagaceae	Leaves	Infusion	Internal	Antypiretics	21	0.33	0.38
				Leaves	Decoction	Internal				
							Pharyngitis			
31	*Cedrela serrata* Royle. (HF-24)	Drawa, Tree	Melliaceae	Stem and bark	Paste	External	Round worms.	54	0.84	0.83
				Root	Juice	Internal	Dyspepsia, Diabetes			
				Leaves	Decoction	External	Refrigerante shampoo			
				Bark	Poultice	Internal	Ulcers			
				Bark	Powder	Internal	Chronic infantile dysentery.			
32	*Celtis caucasica* Willd (HF-72)	Batkaral, Tree	Ulmaceae	Aerial parts	Juice	Internal	Pain, Amenorrhea.	17	0.27	0.45
33	Cichorium intybus L. (HF-142)	Kahsni, Herb	Asteraceae	Roots	Juice	Internal	Antypiretic	33	0.52	0.73
34	*Clematis buchananiana* DC. (HF-84)	Langi, Shrub	Ranunculaceae	Leaves	Paste	External	Eczema	43	0.67	0.75
							Psoriasis (type of skin diseases)			
							Wound			
				Roots	Crushing and wrapping	External	Bleeding			
				Roots	Poultice Juice	External Internal	Swellings, inflammation			
							Peptic ulcers			
35	*Clematis montana* Buch. (HF-118)	Langi, Shrub	Ranunculaceae	Leaves	Extract	Internal	Diabetes	27	0.42	0.33
				Flowers	Decoction	Internal	Cough			
36	*Conyza bonariensis* (L.) Cronq. (HF-51)	Buti, Herb	Asterceae	Aerial parts	Infusion	Internal	Diarrhea	41	0.64	0.77
							Haemorroids			
							Dysentery			
37	*Cuscuta reflexa* Roxb. (HF-131)	Neela dari, Climber	Cuscutaceae	Whole plant	Juice	Internal	Jaundice	21	0.33	0.38
						External	Dandruff			
38	*Dalbergia sissoo* Roxb. (HF-09)	Tahli, Tree	Fabaceae	Stem bark	Juice	External	Eczema	39	0.61	0.77
				Crushed leaves	Juice	Internal	Blood purifier			
				Leaves	Washing	External	Increase hair length			
39	*Debregeasia salicifolia* D.Don Rendle (HF-78)	Sandari, Shrub	Urticaceae	Aerial parts	Paste	External	Eczema, dermatitis	15	0.23	0.41
40	*Desmodium polycarpum* DC. (HF-83)	Mangkit-parang, Shrub	Paplinioaceae	Roots	Juice	Internal	Antypiretic	34	0.53	0.88
							Helminthiasis			
							Haemorroid			
							Antypiretic			
							Cough			
							Cardiac tonic			
							Diuretic			
							Loss of appetite			
							Carminative			
							Diarrhea			
							Dysentery			
41	*Desmostachya bipinnata* L. Stapf. (HF-13)	Dab, Grass	Poaceae	Roots	Tea	Internal	Hypertension	14	0.22	0.17
42	*Dicliptera bupleuroides* Nees (HF-58)	Kirch, Somni, Herb	Acanthaceae	Leaves	Paste	External	Wounds	52	0.81	0.86
							Eczema			
				Leaves	Decoction	External	Tonic			
							Cough			
43	*Dioscorea bulbifera* L. (HF-57)	Herb	Dioscoreaceae	Aerial parts	Juice	Internal	Contraceptive.	41	0.64	0.81
44	*Dioscorea deltoidea*Wall. ex Kunth (HF-50)	Herb	Dioscoreaceae	Rhizome	Taken as raw form	Internal	Insect killer	36	0.56	0.48
							Snake bite			
45	*Duchesnea* Andrews) Focke (HF-84)	Budimewa, Herb	Rosaceae	Fruit	Juice	Internal	Eye Infection	33	0.52	0.61
							Tonic			
46	*Elaeagnus angustifolia* L. (HF-92)	Sinjit, Tree	Elaeagnaceae	Ripe fruits	Boiled	Internal	Pharyngitis, Antypiretic	29	0.45	0.66
				Fruit	Taken as raw form	Internal	Cough Fever			
47	*Elaeagnus umbellata* Thunb. (HF-94)	Chota zaton, Russian olive, Tree	Elaeagnaceae	Leaves	Decoction	Internal	Cough	33	0.52	0.73
				Flowers	Decoction	Internal	Heart diseases.			
				Seeds	Taken as raw form	Internal	Immunity			
				Branch	Exude	External	Toothache			
48	*Eriobotrya japonica* Thumb. Lindler (HF-55)	Loquat, Tree	Rosaceae	Leaves	Poultice	External	Swellings.	44	0.69	0.89
				Fruits	Taken as raw form	Internal	Sedative			
							Emetic			
				Leaves	Infusion	Internal	Relieve diarrhea.			
				Flowers	Infusion	Internal	Refrigerante			
49	*Euphorbia helioscopia* L. (HF-87)	Dhodhal, Dandlion, Herb	Euphorbiaceae	Seeds	Juice	Internal	Cholera	49	0.77	0.72
				Roots	Paste	Internal	Anthelmintic			
50	*Euphorbia wallichii* Hk.f (HF-80)	Dhodhal Dandlion, Herb	Euphorbiaceae	Aerial parts	Latex Juice	Internal	Purgative	42	0.66	0.91
							Dyspepsia			
							Purgative			
							Warts			
							Skin infections			
51	*Ficus carica* L. (HF-54)	Phagwar, Tree	Moraceae	Fruit	Taken as raw form	Internal	Stomachache	52	0.81	0.95
							Haemorroids			
							Cystitis			
							Anemia			
							Constipation			
				Leaves	Latex	External	Wounds			
				Latex	Rubbing	External	Extract thorns from feet or other body organs.			
52	*Ficus palmate* Forssk. (HF-32)	Phaghwar, Anjir, Tree	Moraceae	Fruit	Taken as raw form	Internal	Demulcent	37	0.58	0.84
							Purgative			
							Lungs diseases			
							Refrigerante			
							Cystitis			
				Aerial parts	Paste	External	Freckles			
				Latex		External	Eczeema			
53	*Fragaria nubicola* Lindl. ex Lacaita (HF-87)	Budi meva, Wild Straberry, Herb	Rosaceae	Fruit	Chewed	Internal	Purgative	35	0.55	0.5
							Mouth infection			
							Purgative			
54	*Fumaria indica* (Hausskn.) Pugsley (HF-48)	Papra, Herb	Fumaricaceae	Aerial parts	Juice, Paste	Internal	Antypiretic	48	0.75	0.84
							Skin infection			
							Purify blood			
							Pimples,			
							Constipation			
							Eczima			
55	*Galium aparine* L. (HF-40)	Lainda, Herb	Rubicaceae	Aerial parts	Powder	External	Bleeding	15	0.23	0.31
56	*Galium asperifolium* Wall (HF-80)	Lainda, Herb	Rubicaceae	Aerial parts	Juice	Internal	Diuretic	22	0.34	0.38
							Kidney Infections			
57	*Gerbera gossypina* (Royle) Beauverd (HF-65)	Put Potula, Herb	Asteraceae	Leaves	Paste	External	Skin diseases	37	0.58	0.66
							Bone fracture			
							Wounds and cuts			
							Pain			
58	*Hedera nepalensis* K. Koch (HF-112)	Harbumbal epiphyte	Araliaceae	Leaves	Decoction	Internal	Diabetes	11	0.17	0.13
59	*Heracleum cachemirica* C.B. Clarke (HF-76)	Tukar, Shrub	Apiaceae	Aerial parts	Juice	Internal	Nerve disorders.	18	0.28	0.19
							Nausea			
60	*Heracleum candicans* Wall ex. DC (HF-85)	Herb	Apiaceae	Aerial parts	Tea	Internal	Nerve disorders.	12	0.19	0.14
61	*Hypericum perforatum* L. (HF-02)	Herb	Guttiferae	Flowers	Infusion	Internal	Snake bite	47	0.73	0.61
							Wounds			
							Swellings			
							Rheumatism			
							Sores			
							Ulcers			
62	*Ipomoea carnea* Jac. (HF-142)	Jungli bakhir, Shurb	Convolvulaceae	Leaves	Paste	External	Athlete foot.	33	0.52	0.73
63	*Isodon rugosus* (Wall.ex.Benth.) Codd. (HF-35)	Khwangere, Shrub	Lamiaceae	Leaves	Decoction	Internal	Blood pressure	37	0.58	0.75
							Toothache			
							Fever			
							Rheumatism			
64	*Jasminum mesnyi* Hance (HF-70)	Pili chambali, Shrub	Oleaceae	Leaves	Powder	External	Dandruff	51	0.80	0.67
							Pains			
				Leaves	Chewing	Internal	Mouth ulcers			
				Leaves	Decoction	Internal	Pyorrhea			
				Branches	Ash	External	Headache			
							Joint pain			
				Dried flower	Powder	Internal	Hepatic disorders.			
65	*Juglans regia* L. (HF-71)	Akhrot, Khore, Tree	Juglandaceae	Leave	Decoction	External	Antispasmodic	51	0.8	0.92
				Bark	Rubbing	External	Prophylaxis			
							Lips and gums dye			
				Seeds	Oil	External	Rheumatim			
				Roots and leaves	Powder	External	Antiseptic			
66	*Launaea taraxacifolia* (Willd.) Amin (HF-134)	Hand, Herb	Asteraceae	Whole plant	Taken as raw form	Internal	Diabetes	29	0.45	0.66
							Pain			
67	*Lepidium sativum* L. (HF-136)	Haleon, Herb	Brassicaceae	Seeds	Cooked	Internal	Backache	17	0.27	0.45
68	*Lespedeza juncea* L.f. (HF-06)	Herb	Paplionaceae	Root	Juice	Internal	Diarrhea	26	0.41	0.38
							Dysentery			
69	*Ligustrum lucidum* Ait.f. (HF-111)	Guliston, Shrub	Oleaceae	Aerial parts	Extracts	Internal	Antitumor	23	0.36	0.5
70	*Malvastrum coromandelianum* (L.) Garcke (HF-03)	Herb	Malvaceae	Aerial parts	Decoction	Internal	Kill worms	38	0.59	0.41
							Dysentery			
71	*Melilotus alba* Desr (HF-04)	Herb	Papilionaceae	Leaves	Paste	External	Joint inflammation.	15	0.23	0.3
72	*Mentha royleana* subsp. *hymalaiensis* Briq. (HF-07)	Podina, Herb	Lamiaceae	Leaves	Juice, Powder to make chattni	Internal	Stomach disorder	58	0.91	0.97
							Cough			
							Antypiretic			
							Cholera			
							Emetic			
							Indigestion			
							Gas trouble			
73	*Momordica charantia* L. (HF-129)	Khrella, Climber	Cucurbitaceae	Fruit	Juice	Internal	Diabetes	21	0.33	0.38
							Pain			
				Leaves		External	Swelling			
74	*Momordica dioica* Roxb. ex Willd (HF-152)	Epiphyte	Cucurbitaceae	Roots	Cooked	Internal	Haemorroids	15	0.23	0.17
							Urinary problem			
75	*Myrsine africana* L. (HF-60)	Gorkhan, Chapra, Bebrang, Shrub	Myrsinaceae	Fruits	Powder	Internal	Stomachache			
							Purgative			
							Anthelmintic			
							Parminative			
				Leaves	Decoction	Internal	Blood purifier			
76	*Nepeta erecta* (Boyle ex Benth.) Berth. (HF-50)	Herb	Lamiaceae	Flowers	Juice	Internal	Cough	53	0.83	0.78
				Leaves	Juice	Internal	Blood			
							Pressure			
							Toothache			
							Flu			
							Antypiretic			
							Fever			
77	*Nepeta laevigata* D.Don Hand (HF-53)	Herb	Lamiaceae	Fruit	Infusion	Internal	Dysentery	17	0.27	0.22
78	*Nerium oleander* L. (HF-37)	Kanair, Tree	Apocynaceae	Leave	Paste	External	Cutaneous eruption	46	0.72	0.98
				Leave	Decoction	Internal	Wounds			
							Swelling			
				Bark	Decoction	Internal	Eczema, leprosy			
				Roots	Powder	Internal	Abortion			
				Roots	Paste	External	Scorpion sting, snakebite.			
79	*Oenothera rosea* L.Her. ex. Ait (HF-91)	Buti, Herb	Onagraceae	Leaves	Infusion	Internal	Hepatic pain	45	0.7	0.64
							Kidney disorders			
80	*Opuntia dillenii* Haw. (HF-142)	Thor, Shurb	Cactaceae	Whole plant	Paste	External	Joints pain	33	0.52	0.5
81	*Parthenium hysterophorus* L. (HF-07)	Herb	Asteraceae	Root	Decoction	Internal	Eczema	35	0.55	0.59
							Dysentery			
82	*Pimpinella stewartii* Dunn. E.Nasir (HF-08)	Tarpakki, Herb	Apiaceae	Fruit	Taken as raw form	Internal	Stomachache	12	0.19	0.3
83	*Pinus roxburgii* Roxb (HF-94)	Chir, Tree	Pinaceae	Leaves bark Powder	Juice	Internal	Dysentery.	58	0.91	1.13
				Resin	Poultice	Internal	Ulcer			
							Tumor			
							Bleeding			
							Cough			
							Snake bite			
							Wound			
84	*Pinus wallichiana* A.B. Jackson (HF-58)	Biar, blue pine, Tree	Pinaceae	Resin	Poultice	External	Wound	42	0.66	0.84
85	*Pistacia chinensis* ssp.* Integerrima* (J. L. Stewart) Rech. f. (HF-22)	Kangar, Tree	Anacardiaceae	Stem gum	Powder	Internal	Dysentery.	43	0.67	0.91
	
				Bark	Paste	External	Cracked heels			
							Wound			
86	*Plantago lanceolata* L. (HF-88)	Ispgol, Herb	Plantaginaceae	Leaves	Paste	External	Wound	53	0.83	0.91
				Seeds	Extract	Internal	Toothache			
							Purgative			
							Hemostatic			
							Dysentery			
87	*Poa nepalensis* Walls ex. Duthie. (HF-131)	Grass	Poaceae	Leaves	Decoction mixed with water	External	Anti-lice	29	0.45	0.42
88	*Podophyllum emodi* Wall ex Royle (HF-87)	Banhakri Herb	Podophyllaceae	Root	Extract	Internal	Purgative,	48	0.75	0.83
							Liver			
							Stomachache			
89	*Polygonatum multiflorum* L. Smith (HF-78)	Herb	Liliaceae	Leave	Paste	External	Wound	17	0.27	0.19
90	*Polystichum squarrosum* (D. Don) Fée (HF-114)	Gha, Fern	Dryopteridaceae	Root	Decoction	Internal	Pyloricdisease	13	0.2	0.3
91	*Prunella vulgaris* L. (HF-63)	Herb	Lamiaceae	Seeds	Taken as raw form	Internal	Purgative	58	0.91	0.98
							Antipyretic			
							Tonic			
							Eye sight weakness			
							Astama			
							Heart diseases			
							Inflammation			
							Diuretic			
92	*Prunus armeniaca* L. (HF-24)	Hari, Khubani, Apricot, Tree	Rosaceae	Fruit	Taken as raw form	Internal	Purgative	31	0.48	0.39
				Seed	Oil	External	Softening effect on the skin.			
93	*Prunus domestica* L. (HF-31)	Lucha, Alu bukhara, Tree	Rosaceae	Fruit	Taken as raw form	Internal	Miscarriage	34	0.53	0.84
							Irregular menstruation debility			
94	*Prunus persica* L.Batch (HF-49)	Aru, Peach, Tree	Rosaceae	Leaves	Juice	Internal	Chest infection	44	0.69	0.88
							Gastritis			
							Whooping cough			
							Bronchitis			
							Intestinal Anthalmatic			
							Anthalmatic for cattles			
95	*Pteris cretica* L.(HF-158)	Cretan brake, Fern	Pteridaceae	Leaves	Paste	Internal	Wound	9	0.14	0.17
96	*Punica granatum* L. (HF-22)	Druna, Tree	Punicacea	Fruit	Taken as raw form	Internal	Cough, tonic	52	0.81	1
				Leaves	Juice	Internal	Dysentery			
				Bark stem and root	Decoction	Internal	Mouthwash			
							Anthelmintic for tapeworms			
							Expectorant			
97	*Pyrus malus* L. (HF-26)	Saib, Tree	Rosaceae	Fruit	Juice, paste	Internal	Rheumatism	46	0.72	0.81
							Hypertension			
							Tonic for vigorous body			
							Fracails			
							Strengthen bones			
							Constipation			
98	*Pyrus pashia* Ham.ex.D.Don (HF-17)	Butangi, Tree	Rosaceae	Fruit	Taken as raw form	Internal	Eye dark circles	49	0.77	0.95
99	*Quercus baloot* Griff (HF-90)	Rein, Shah baloot, Oak, Tree	Fagaceae	Bark	Powder	Internal	Asthma	43	0.67	0.86
				Nut	Decoction	Internal	Urinary problems, cough, cold.			
100	*Quercus dilatata* Royle (HF-68)	Oak, Barungi, Tree	Fagaceae	Fruit	Powder	Internal	Tonic	47	0.73	0.36
				Bark	Decoction	Internal	Dysentery			
101	*Quercus incana* Roxb. (HF-49)	Rein, Ban, Rinji, Tree	Fagaceae	Bark	Powder	Internal	Asthma	41	0.64	0.95
							Antypiretic			
							Rheumatism			
							Backache			
							Cough			
102	*Ranunculus muricatus* L. (HF-76)	Herb	Ranunculaceae	Aerial parts	Cooked	Internal	Asthma	14	0.22	0.19
103	*Robinia pseudoacacia* L. (HF-83)	Kikar, Tree	Papilionaceae	Bark	Chewing	External	Toothache	31	0.48	0.8
104	*Rosa brunonii* Lindl. (HF-121)	Chal, Tarni, Musk Rose, Shrub	Rosaceae	Flower	Decoction	Internal	Constipation	49	0.77	0.84
				Flowers	Powder	Internal	Diarrhea			
							Heart tonic			
							Eye diseases			
							Eczema			
							Wounds			
105	*Rubus fruticosus* Hk f. non L (HF-50)	Garachey, Shrub	Rosaceae	Leaves	Infusion	Internal	Diarrhea	57	0.89	0.98
							Antypiretic			
				Bark	Soaking	Internal	Diabetes			
106	*Rubus niveus* Thunb. (HF-100)	Garachey, Shrub	Rosaceae	Leaves	Extract	External	Urticaria (skin disease)	32	0.5	0.59
				Leaves	Powder	Internal	Diarrhea			
							Diuretic			
							Antypiretic			
				Root	Decoction	Internal	Dysentery			
							Colic pains			
							Whooping coughs			
							Diarrhea			
107	*Rumex dentatus* L. (HF-101)	Jangli Palak Herb	Polygoneaceae	Leaves	Paste	External	Wound	41	0.64	0.59
				Roots	Paste	External	Eczema			
108	*Rumex hastatus* L. (HF-44)	Khatimal, Shrub	Polygonoceae	Roots	Juice	Internal	Asthma	32	0.5	0.64
							Weakness in cattle			
							Antypiretic			
							Cough			
109	*Salix acmophylla* Boiss. (HF-85)	Beens, Bed, Gaith, Tree	Salicaceae	Leaves	*Paste, boiled with Robinia pseudoacacia and Cotula anthemoids*	Internal	Boils	51	0.8	0.98
				Branch	Chewing	Internal	Hernia			
							Antypiretic			
							Joints Inflammation			
							Stomachache			
110	*Salix denticulata* Andersson (HF-79)	Terik, Jangali Bed, Tree	Salicaceae	Stem and root bark	Boiled	Internal	Headache	34	0.53	0.39
							Antypiretic			
							Paralysis			
				Leaves, branches	Paste	External	Itching			
							Eczema			
111	*Salvia hians* Royle (HF-107)	Herb	Lamiaceae	Leaves	Juice	Internal	Cough	31	0.48	0.66
							Fever			
							Anxiety			
112	*Salvia lanata* Roxb. (HF-51)	Herb	Lamiaceae	Leaves	Poultice	External	Eczema	27	0.42	0.48
							Wound			
113	*Salvia moorcroftiana* Wall. Ex Benth (HF-59)	Kaljari, Herb	Lamiaceae	aerial parts	Juice	Internal	Diarrhea	51	0.8	0.89
							Gas trouble			
							Cough			
							Stomachache			
114	*Sambucus wightiana* Wall. ex Wight & Arn. (HF-52)	Gandala, Herb	Caprifoliaceae	Fruit	Taken as raw form	Internal	Stomachache	19	0.3	0.5
							Anthelmintic			
115	*Sapindus mukorossi* Gaertn. (HF-79)	Ritha, Soap nut, Tree	Sapindaceae	Seeds	Powdered	External	Anthalmantic	47	0.73	0.77
				Fruits	Rubbing	External	Boils			
116	*Sarcococca saligna* D. Don Muell (HF-66)	Bansathra, Shrub	Buxaceae	Leaves and shoots	Decoction	Internal	Joints pain	23	0.36	0.23
							Blood purifier			
							Purgative			
				Leaves	Powder	External	Burns			
				Root	Juice	Internal	Gonorrhea.			
117	*Saussurea candolleana* Wall. Ex. D.C Clarke (HF-84)	Herb	Asteraceae	Roots	Extract	Internal	Tonic	23	0.36	0.28
118	*Skimmia laureola* DC. Sieb (HF-08)	Nazar Panra, Tree	Rutaceae	Leaves	Powdered	External	Smallpox, anthalmatic, colic.	48	0.75	0.59
119	*Smilax glaucophylla* Klotroch (HF-115)	Epiphyte	Smilicaceae	Aerial parts	Infusion	Internal	Carminative	32	0.5	0.55
							Dog bite			
							Spasm			
							Antypiretic			
120	*Sophora mollis* (Royle) Baker (HF-56)	Buna, Sakina, Shrub	Paplionaceae	Flowers	Powder	External	Pimples	21	0.33	0.36
							Wound			
							Inflammation			
							Sun burns			
121	*Swertia ciliate* G.Don B. L. Burtt (HF-75)	Herb	Gentianaceae	Aerial part	Decoction	Internal	Cough	48	0.75	0.88
							Cold			
							Antypiretic			
122	*Taraxacum officinale* F.H. Wigg (HF-102)	Handh, Herb	Asteraceae	Roots	Decoction	Internal	Jaundice	56	0.88	0.92
				Leaves	Cooked	Internal	Swellings			
							Diuretic			
							Tonic			
123	*Themeda ananthra* Nees ex Steud.Anderss. (HF-39)	Grass	Poaceae	Aerial parts	Poultice	External	Backche	41	0.64	0.5
				Leaves	Decoction	Internal	Blood purifier			
124	*Thymus liniaris* Benth. Subsp. *Liniaris* Jalas (HF-105)	Herb	Lamiaceae	leaves and flowers	Powder	Internal	Strengthen teeth and gum	32	0.5	0.64
							Bleeding			
				Flower	Grounded seeds of *Carum carvi* with flowers	Internal	Digestion			
125	*Trichodesma incanum* (Bunge) A. DC. (HF-145)	Handusi booti Herb	Borangniceae	Leaves	Boiling	Internal	Flu	31	0.48	0.48
							Cough			
126	*Trichodesma indicum* (L.) R. Br.(HF-178)	Handusi, Herb	Boraginaceae	Whole plant	Cooked	Internal	Backache	41	0.64	0.5
					Internal		Weakness			
					External		Kidney stone			
127	*Valeraina jatamansi* Joes (HF-77)	Herb	Valerianaceae	Aerial parts	Oil	Internal	Constipation	19	0.3	0.41
128	*Verbascum thapsus* L. (HF-107)	Gider tabacoo Herb	Valerianaceae	Roots	Decoction	Internal	Toothache	17	0.27	0.25
							Convulsions			
							Bleeding gum			
129	*Vibernum nervosum* D.Don (HF-78)	Taliana, Shrub	Caprifoliaceae	Fruit	Taken as raw form	Internal	Stomachache	15	0.23	0.3
							Anemia			
130	*Viburnum cotinifolium* D. Don (HF-95)	Taliana, Shrub	Caprifoliaceae	Fruit	Taken as raw form	Internal	Purgative	31	0.48	0.33
							Blood Purifier			
				Leaves	Extract	Internal	Menorrhagia			
131	*Viburnum grandiflorum* Wall.ex.DC (HF-47)	Guch, Shrub	Caprifoliaceae	Seed	Juice	Internal	Typhoid	25	0.39	0.2
							Whooping cough			
132	*Vincetoxicum hirundinaria* Medicres (HF-109)	Herb	Asclepidaceae	Aerial parts	Decoction	Internal	Boils	48	0.75	0.8
							Pimples			
133	*Viola canescens* Wall.ex Roxb. (HF-108)	Banafsha. Herb	Violaceae	Leaves	Juice	Internal	Cough	51	0.8	0.84
							Jaundice			
							Antypiretic			
							Fever			
134	*Viola pilosa* Blume (HF-11)	Banafsha. Herb	Violaceae	Leaves	Decoction	Internal	Pain	47	0.73	0.81
							Antypiretic			
							Stomach ulcer			
135	*Zanthoxylum armatum* DC. Prodr (HF-88)	Timbar, Shrub	Rutaceae	Fruit, branches	Juice	Internal	Carminative	60	0.94	1.13
							Cholera			
							Stomachache			
							Gum, toothache			
							Indigestion			
							Haemorroids			
							Stomachache			
				Seed	Powder	Internal	Stomachache			
							Toothache			
							Gums problems			
					Chewed	Internal	Antipyretic			
							Stomachic			
							Diuretic			
136	*Ziziphus nummularia* (Burm. f.) Wight & Arn. (HF-88)	Ber, Tree	Rhamnaceae	Fruit	Decoction	External	Dandruff	51	0.8	0.98
				Bark	Mixed with Milk and honey	Internal	Diarrhea and dysentery			

Key words: FC = Frequency of citation; RFC = Relative Frequency of Citation; UV = Use Value

Interviews were carried out complying with the ethics guidelines commonly followed in ethnobotanical studies, and the informants’ written consent was obtained prior to the interviews. In order to ensure that the information was as unbiased as possible, we tried to avoid the presence of other people during the interviews. Participant observation was also used in order to better interpret and analyze the data reported by informants. The information collected concerned both diseases (the most frequent ones, ways of classifying and diagnosing them, etc.) and medicinal plants (local names, indications of use, plant parts used, places/methods/rituals of gathering, utilization and administration).

Voucher specimens were gathered using the informants’ indications, prepared according to standard taxonomic methods, and conserved in our lab for future reference. For plant identification, we consulted the Flora of Pakistan (www.eflora.com). Botanical nomenclature is presented in accordance with the International Plant Name Index (IPNI) (www.ipni.org).

### Research hypothesis

The hypothesis for the present study was, older people know more uses of plants than younger, formal education is not predictive of knowledge level about plants, and men tend to know more plant species/users than women. Species of plants are of unequal usage/importance to people. Across the range of species, its importance will vary, even among communities with the same cultural origin. In addition, it was also hypothesized that closely related plants are exploited in the treatment of almost similar diseases in cultures that are not much related thus are more likely to be independent discoveries of similar plant compounds and disease mechanisms.

### Quantitative ethnobotanical data analysis

To determine whether a statistically significant correlation exists between the numbers of plants mentioned and the informant’s age, we used the Spearmann test. The Mann-Whiney U and Kruskal-Wallis tests were used to find significant differences between two and among 5 groups related groups, respectively, all set at 0.5 alpha level of significance.

Some quantitative indices commonly adopted in ethnobotanical studies were used to analyze the data collected through the interviews [29]. Relative frequency of citations (RFC) and use value (UV) was used to access relative importance of plant species cited by informants.

Frequency of citations was estimated as

$$RFC=\frac{FC}{N}$$

Where FC is the number of informants reporting the use of a particular species and N is the total number of informants.

Use value [30] was estimated as

$$UV=\sum \frac{Ui}{N}$$

Where Ui is the number of uses mentioned by each informant for a given species and N is the total number of informants.

### Informant consensus factor (ICF)

Informant consensus factor was used to identify the most trusted healing plants for those disease categories that were claimed to be most common in the area following the approach of [31] by using the following formula:

$$ICF=\frac{Nur-Nt}{Nur-1}$$

Where Nur is the number of use-reports in each disease category and Nt is number of species used.

### Jaccard index (JI)

JI was calculated in order to compare data reported in our study with previously published data collected from neighboring regions by using the following formula

$$JI=\frac{c}{a+b-c}\times 100$$

Where

a = number of species found only in area A
b = number of species found only in area B
c = number of species common to both areas

## Results and discussion

### Diversity of medicinal plants in the studied area

A total of 136 medicinal plant species belonging to 98 genera and 45 families were reported “Table 1”. They represent about 19–23% of the pool of medicinal plants that constitute the pharmaceutical ethnoflora in alpine areas of Pakistan [32]. The most represented botanical family was Asteraceae (14 species, 10%), followed by Lamiaceae (11 species, 8.1%), Fabaceae and Rosaceae (5 species each, 3.7%), and Ranuncolaceae (4 species, 2.9%). The other 41 families contributed with less than 4 species; among these 41 families, 26 (58% of all cited families) were represented with only 1 species. These results were in accordance with other ethnobotanical studies carried out in Pakistan [33–35] and were not surprising. In fact, Asteraceae, Lamiaceae, Fabaceae, Rosaceae are large, mostly cosmopolitan families that are known worldwide to be medicinal; for example, Asteraceae and Lamiaceae are rich in essential oils and are widely used in popular medicine around the world [36–38]. Rosaceae is rich in phenols, a group of substances that play an important role as anti-oxidants in the human diet [38]. Ranuncolaceae is rich in active secondary metabolites and have a high number of pharmacological properties [39, 40]. On the other hand, all of these botanical families contain some plants commonly found in the Pakistan flora. In terms of the life form, most of the used species were herbaceous (55%) followed by trees (26%), shrubs (17%), and climbers (2%). As is generally found in alpine areas (see for example [24]), the use of herbaceous species is more frequent than the use of perennial woody species. This finding can be related to the composition and the structure of the vegetation of high altitude areas, where tree growth is made difficult by climatic conditions; on the other hand, herbal preparation methods and extraction of active metabolites are easier to carry out with herbaceous plants than woody materials [41].

#### Herbal drug preparation and utilization

**Fig 2:**
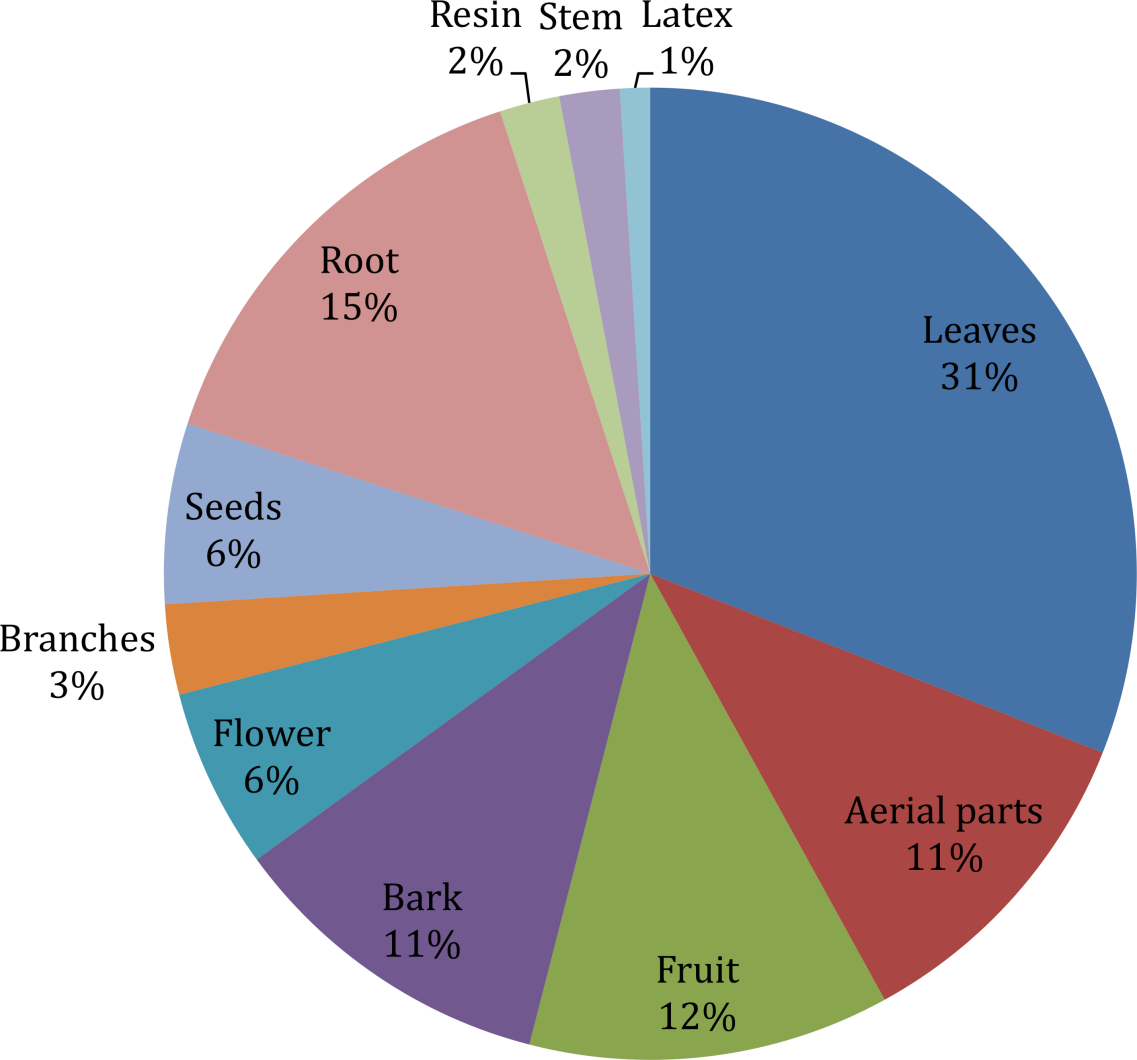
Plants parts use in herbal recipes.

Different plant parts are used differently in herbal medicines depending upon the cultural knowledge and availability of those parts to local inhabitants. Similarly to what reported in other studies [42, 43], leaves (31%) were the most common plant part used in herbal preparations followed by roots (15%) and fruits (12%) “Fig 2”. Leaves are the plant part directly involved in photosynthesis, producing several active compounds like sugars but also storing important secondary metabolites like terpenes, alkaloids, cyanogenic glycosides; these chemical substances are involved in complex plant-to-plant and plant-to-animal relations including defense against a variety of pests and predators and allelopathy and most of these substances are of medicinal value. Roots were the favored plant part in many other cases, possibly because hypogeous organs normally have a high content of secondary metabolites [44]. As reported in previous studies conducted in other areas of Pakistan [45, 46], the utilization of fruits is found to play an important role in the local pharmacopoeia; this can be related to the importance that some wild edible fruits assume as nutraceuticals and in preventing nutritional deficiencies. In 67 cases, different parts of the same plant were used to treat different diseases, for example, roots of *Berberis lyceum* were used internally for the treatment of chronic diarrhea, piles, diabetes, pustules and scabies while externally these were used to heal fractured bone and swellings.

**Fig 3:**
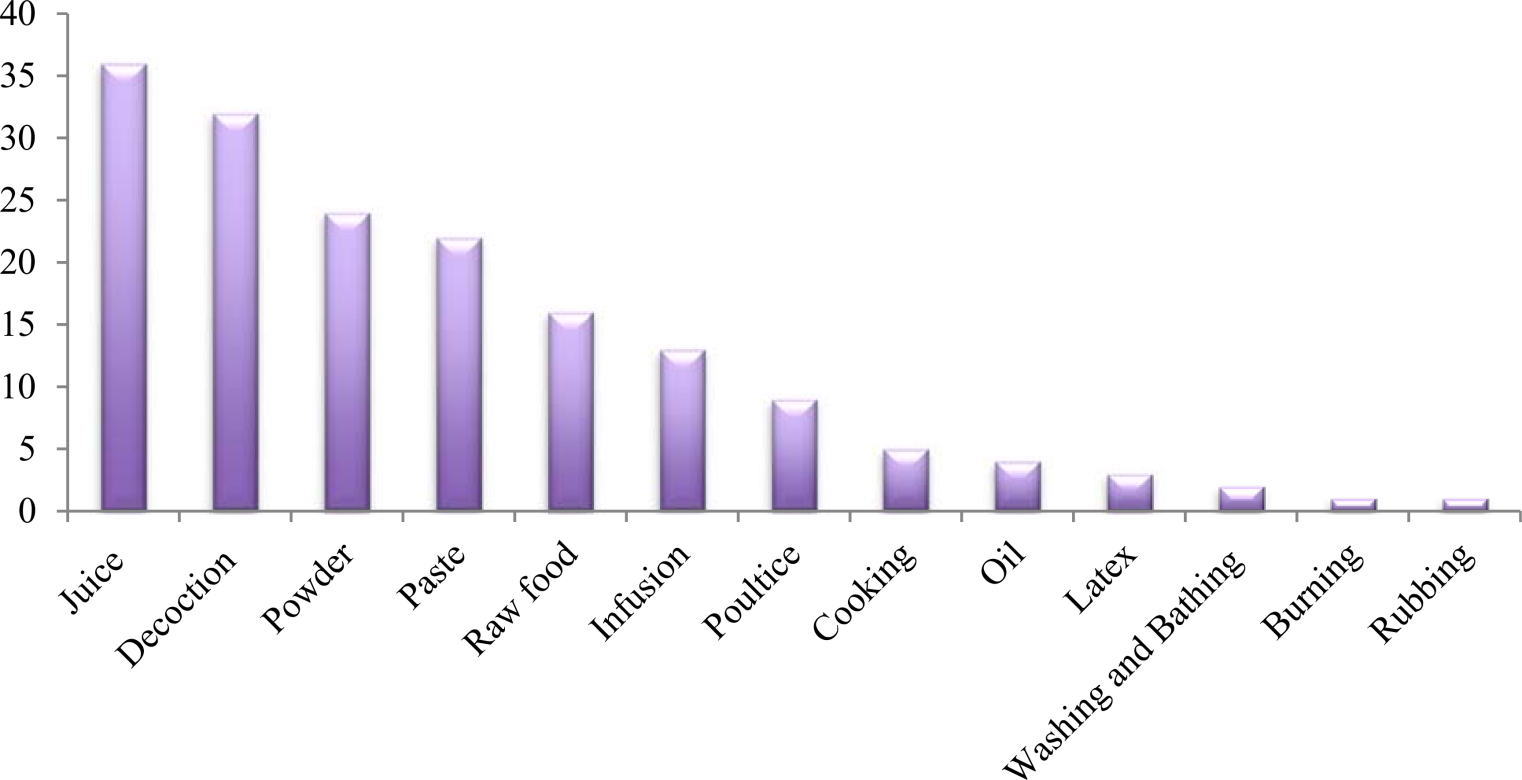
Method of preparation of herbal recipes.

The main method of preparation was decoctions (26 species) followed by juice and powder (24 species each), paste (22), chewing (eating in raw form as salad or fruits) (16 species), extract (11 species), infusion (10 species) and poultice (8 species) “Fig 3”. These results were in agreement with those reported in other studies [47–51]. Quantity and dosage of medicinal drugs were found to differ with age, state of health of the patient and severity of the treated disease for example decoction of *Elaeagnus umbellata* for heart diseases if someone is suffering from chronic cardiac problem then he must use this decoction for long time as compare to the patient who was suffering acute cardiac problems. *Ficus carica* is very effective in constipation, for child two to three fruits were effective but for adult four to five fruits were effective for the relief of constipation. The high usage of freshly prepared juice in ethnomedicines was an indication of the high abundance of medicinal plants in the study areas that can be freshly available and harvested anytime for use. The other reasons for the repeated use of fresh plant juice could be the facts that the drying process contributes to the loss of volatile oil and that proteins become denatured at a high temperature. The measurements used to determine the dosages are not standardized and depend on the age and physical appearance of the patient, sociocultural explanation of the illness, diagnosis, and experience of the individual herbalist. Usually, the treatment of the patient is completed within a single day or a couple of days. When patients did not show any indication of improvement from their sickness following the completion of treatment, the physician referred them to a modern health center in an urban area for further examination [46, 52, 53].

Most of the cited plant drugs were administrated (82) as they are, but in some herbal preparations (50) they were mixed with other ingredients like milk, water, honey, oil or butter to treat specific diseases. Few species (4) were used in combination with other herbs, for example, leaves of *Salix acmophylla* boiled with *Robinia pseudoacacia* (leaves) and *Cotula anthemoids* (leaves) to treat fever and *hernia*. As hypothesized in other studies it is possible that interactions among different species involve strengthening of therapeutic effects as well as attenuation of toxicity or of adverse effects of some plants composing the mixture. Most of the herbal preparations were taken internally (68%) while few were also used externally (32%).

Most plants (84; 61.8%) were used in only one (42) or two (42) categories. *Berberis lyceum* and *Prunella vulgaris* were the most versatile species because their use was cited in 8 different medicinal categories followed by *Albizia lebbeck* (7 categories), *Desmodium polycarpum*, *Jasminum mesnyi* and *Pinus roxburghii* (6 categories). Medicinal plants were mainly used to treat digestive disorders (63 species), followed by skin problems (59 species) and respiratory problem.

Among the subcategories mentioned (corresponding to detailed medicinal uses), cough was treated with the highest number of different plants (28 species), followed by eczema (26), constipation (24), and fever (21). Thirty-nine subcategories (36%) were treated with only one species: for example, typhoid was treated with juice extracted from the seeds of *Viburnum grandiflorum*, psoriasis with a paste of leaves of *Clematis buchananiana*, gonorrhea with juice extracted from the roots of *Sarcococca saligna*, and athlete’s foot with a paste of leaves of *Ipomoea carnea*. According to Albuquerque [54], the presence of a large number of species for the same medicinal use would result in maintenance of that use, i.e., it's resilience in the local ethnomedical system. On the other hand, categories associated with only one species could be considered more vulnerable to perturbations. As observed by Numa [55] any change leading to the disappearance of that single species could induce a change in local medical practices, such as searching for alternatives for treating that specific illness.

**Fig 4:**
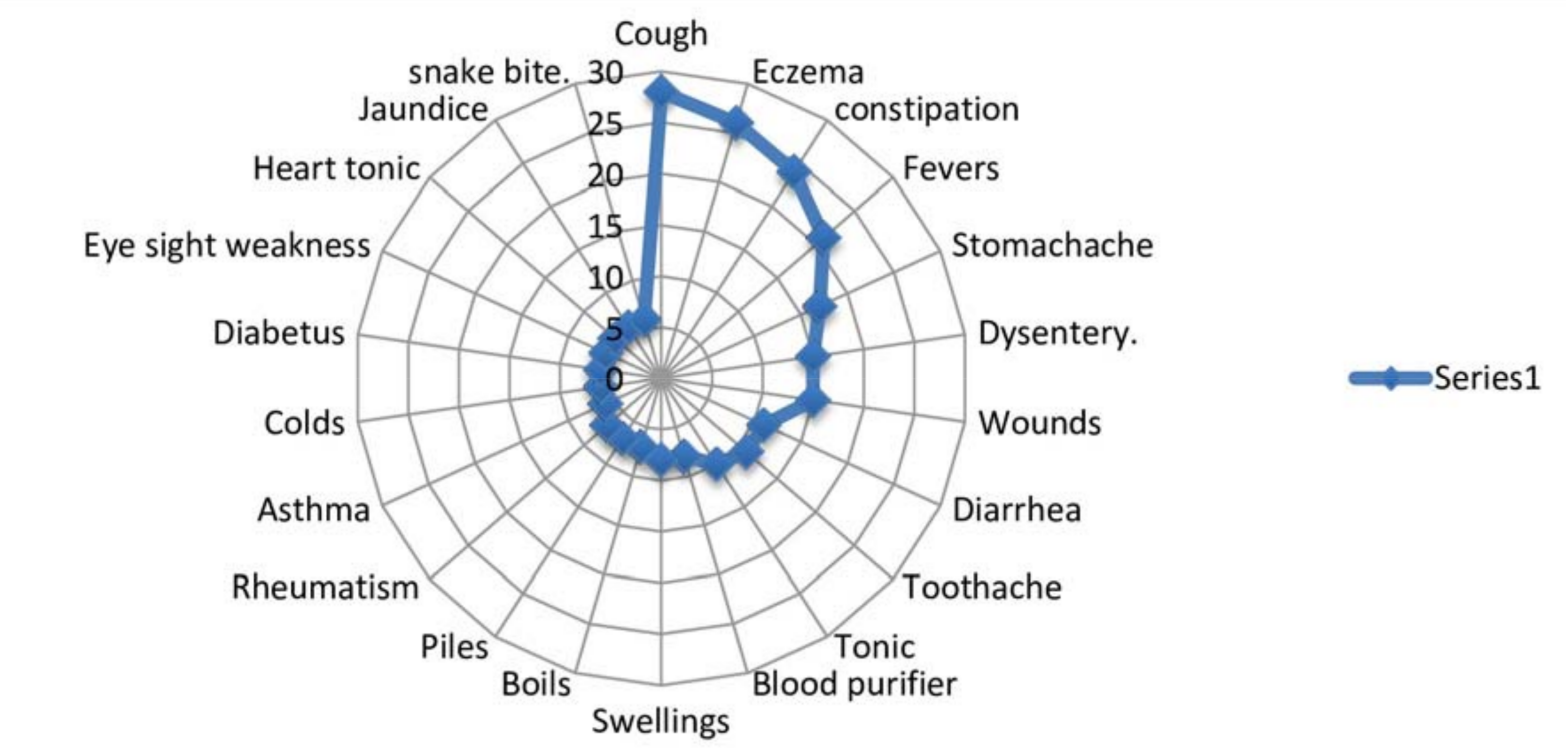
Different disease treated by the plants in the study area.

According to other studies conducted in neighboring areas [56–58] and in our research most species (28) were used to treat a cough and related respiratory problems. Climatic factors, such as rarefied air due to the reduction in oxygen found at high altitudes and low-temperature regime experienced throughout the year, combined with high demographic density and poor housing conditions, could explain the high prevalence of contagious diseases. Moreover, the lack of awareness about personal hygiene and cleanliness practices is probably responsible for the high number of plants (26) reported to treating skin problems “Fig 4”.

### Quantitative indices

The relative frequency of citation (RFC) shows the local importance of each species with reference to informants who cited these medicinal plant species. We found that the species with the highest RCF values in the study area were *Zanthoxylum armatum* (0.94), *Berberis lyceum* (0.92), *Ajuga bracteosa* (0.91), *Prunella vulgaris* (0.91), *Pinus roxburghii* (0.91), *Adiantum capillus-veneris* (0.89), *Rosa brunonii* (0.89), *Albizia lebbeck* (0.89), *Cedrella serrata* (0.84), *Punica granatum* (0.81) and *Jasminum mesnyi* (0.80) “Table 1”. Use value (UV) is an index commonly adopted to measure the relative importance of a given species known within a local community. It is high when many uses are reported (i.e., when a species is frequently quoted by informants in the treatment of various diseases) and approaches zero when few uses are mentioned. In this study, the highest use values were reported for *Berberis lyceum* and *Ajuga bracteosa* (1.13 each), *Abies pindrow* (1.03), *Prunella vulgaris* and *Adiantum capillus-veneris* (1.00 each), and *Desmodium polycarpum* and *Pinus roxburghii* (0.98 each) “Table 1”. Because these plants were native and recorded as very common in the area, they combine the cultural value pointed out by our study with a high ecological and landscape importance. These findings were also in accordance with the “appearance hypothesis” [59]: the more common a plant is in an area, the greater the folk knowledge about its use; that is, more common plants would allow local people to have more experience of their properties and consequently would have a greater probability of being introduced into the local culture. UV is dynamic and changes between different areas and even within the same area over time. For example, *P*. *roxburghii* was found to be one of the least cited medicinal plants (UV = 0.01) in the Sudanhoti district (AJK), although the use of this plant for timber, fuel, and construction was well known in the area [59]. On the other hand, *Achyranthes aspera* and *Momordica charantia* had among the highest UVs in the study by [60], while showing low UVs (0.38) in our study. According to some authors [29, 61, 62], medicinal plant species with high RFCs and UVs should be tested to assess and prove their pharmacological activity. On the other hand, plants with low UVs are not necessarily unimportant, but their low values indicate that locals are not aware of their uses, and this could put knowledge about their use at the risk of not being transmitted [19].

### Informant consensus factor

The informant consensus factor (ICF) calculated for each category ranged from 0.69 to 0.90 with a mean value of 0.87. The highest values were recorded for digestive system diseases (ICF = 0.90), muscular and skeletal system diseases (ICF = 0.89), and mouth/pharynx diseases and diabetes (ICF = 0.86 each). The lowest ICF value was found for the hair care category (0.69) “Table 2”. ICF values are influenced by the number of informants and are more significant when calculated for uses cited by many informants. In general, ICF values were high in our study, revealing that the informants tend to agree on which plants to use in the treatment of common illnesses. According to Heinrich [63], high ICF can help in identifying potentially effective medicinal plants. It must be highlighted that in our study the highest agreement level was recorded for diseases reported as the most widespread in rural communities of the Poonch district and other areas of Pakistan [64]. The prevalence of gastrointestinal disorders in the study area may be attributed to the low availability of hygienic food and drinking water. According to the District Census Report carried out in 1998, the population with access to safe drinking water inside the house was 16.28%; in 2005 the water access, although improved (21%), was still strongly lacking. This situation was compounded by the devastating earthquake, in which several water-supply schemes were damaged and the drinking water supply was obtained from contaminated surface water sources [65].

**Table 2:** Informant consensus factor (ICF) of Pearl Valley Tehsil Rawalakot, District Poonch AJ&K.

S. NO.	Diseases	Detailed categories	Ntax	Nur	ICF	Most Used Plants
1	Colds, respiratory tract diseases	Asthma, breathing difficulties, bronchitis, chest pain, cough, expectorant, Flu, lungs diseases	41	236	0.83	*Mentha royleana*, *Polygonatum multiflorum*, *Punica granatum*, *Pyrus pashia*, *Salvia moorcroftiana*, *Prunella vulgaris*
2	Digestive system disorders	Anthelmintic, Cholera, constipation, diarrhea, dysentery, dyspepsia, flatulence, gastritis, jaundice, liver and bile diseases, nausea, stomachache, typhoid, vomiting	114	1162	0.90	*Mentha royleana*, *Zanthoxylum armatum*, *Berberis lyceum*, *Eriobotrya japonica*, *Punica granatum*, *Ziziphus numelaria*, *Artemisia absinthium*
3	Urinary and sexual-reproductive system diseases	Abortion, amenorrhea, aphrodisiac, contraceptive, diuretic, gonorrhea, irregular menstruation, kidney diseases, kidney stones, leucorrhoea, menorrhagia, miscarriage, urinary tract diseases	16	91	0.83	*Aesculus indica*, *Prunus domestica*, *Bergenia ciliata*, *Galium asperifolium*, *Oenothera rosea*, *Eriobotrya japonica*,
4	Muscular and skeletal system diseases	Antispasmodic, bone fracture, backache, body and joint inflammations, cramps, muscular pains, joint pains, paralysis, rheumatism	11	100	0.89	*Hypericum perforatum*, *Juglans regia*, *Pyrus malus*, *Pyrus malus*
5	Nervous system diseases	Convulsions, depression, general pain, headache, nervous problems, sedative, toothache	16	86	0.82	*Juglans regia*, *Pyrus malus*, *Heracleum candicans*
6	Circulatory system diseases	Bleeding, hemorrhoids, hearth diseases, hearth tonic, hypertension, pressure	6	25	0.79	*Rosa brunonii*,*Oenothera rosea*,*Viola canscens*, *Adiantum capillus-veneris*, *Desmostachya bipinnata*,*Pyrus malus*
7	Blood and lymphatic system	Anemia, blood purification	15	76	0.81	*Dalbergia sissoo*, *Rosa brunonii*, *Berberis lyceum*,*Vibernum nervosum*,
8	Skin diseases, burns and wounds	Athlete foot, boils, burns, carbuncles, cracked heels, cuts and wounds, dandruff, eczema, eruption, freckles, itching and allergy, leprosy, leukoderma, measles, not specified skin problems, pimples, scabies, skin parasites, smallpox, sun burns, swellings, ulcers, urticaria, warts	47	306	0.85	*Fumaria indica*, *Adiantum incisum*, *Euphorbia wallichii*, *Gallium asperifolium*, *Rosa brunonii*
9	Fever	Fever	32	138	0.77	*Smilax glaucophylla*, *Abies pindrow*, *Castanea sativa*, *Cichorium intybus*, *Elaeagnus angustifoli*, *Quercus incana*
10	Mouth-pharynix diseases	Gum infection, mouth infection and inflammation, pyorrhea, sore throats, strengthening of spongy gums, teeth cleaning	42	306	0.86	*Berberis aristata*, *Juglans regia*, *Thymus liniaris*, *Castanea sativa*,*Pistacia chinensis ssp*. *Integerrima*, *Zanthoxylum armatum*, *Ajuga bracteosa*
11	Antidote	Dog bite, scorpion sting, snake bite	8	31	0.77	*Nerium oleander*, *Dioscorea deltoidea*, *Hypericum perforatum*
12	Ear and eye diseases	Earache, eyes diseases, eye sight weakness	38	186	0.80	*Rosa brunonii*, *Androsace rotundifolia*,*Bergenia ciliata*
13	Hair care	Hair washing, hair conditioner	9	27	0.69	*Juglans regia*, *Poa nepalensis*
14	General weakness in men and animals	Weakness, loss of appetite, tonic	45	258	0.83	*Fumaria indica*, *Asparagus filicinus*,*Castanea sativa*, *Viola canscens*, *Trichodesma indicum*, *Punica granatum*, *Berberis lyceum*, *Ligustrum lucidum*
15	Diabetes		6	36	0.86	*Berberis lyceum*, *Clematis montana*, *Rubus fruticosus*,
14	Others	Demulcent, body temperature, cooling agent, immunity, tumor	15	58	0.75	*Pinus roxburgii*, *Ligustrum lucidum*, *Cedrela serrata*, *Viola canscens*, *Ficus palmate*, *Elaeagnus umbellata*

Key words:Ntax = Number of taxa; Nur = Number of use Reports; ICF = Informant consensus factor

### Medicinal plant knowledge

Each informant mentioned on average 3.75 (± 2.96; minimum: 1; maximum: 15) species and 11.84 (± 10.52; minimum: 2; maximum: 39) different uses. Many studies have shown that age and gender are two important factors to consider when evaluating the distribution of knowledge within a group of informants [66, 67]. However, very few studies have analyzed the effect of these variables on the distribution of ethnomedicinal knowledge in Pakistan (see, for example, Ahmad *et al*. [48] and none of them used statistical analyses for validation of the collected data. 58% of the 64 informants reporting the use of medicinal plants were over 40 years old (37 informants); of these, 17 (26.5% of the informants) were aged between 41 and 50 while 20 (31.5%) were over 50 (Table 1). Spearman’s correlation analysis showed significant positive differences between the age and the number of both number of mentioned species (rs = 0.49; *p* < 0.05) and different uses (rs = 0.45; *p* < 0.05), indicating that there is a trend of older people being more knowledgeable that younger people “Table 3”. When we analyzed the age groups individually, significant differences were observed only among some groups “Table 3”. The youngest informants (from the ages of 19 to 30) knew fewer species (1.33 ± 0.55) and uses (3.00 ± 1.11) than other groups. The informants belonging to the 41–50 and 51–60 age groups cited a higher number of species and uses than the other groups “Table 3”. This trend is widely observed in literature; on the other hand, elderly people are expected to have accumulated more experiences about the uses of medicinal plants for a longer period of time than others. It might also be hypothesized that young people are not interested in learning the use of medicinal plants as a consequence of the socio-economic transition occurring in Pakistan. Comparing the knowledge held by men and women, men had much higher knowledge on medicinal plants (*Z* = 3.20; *p* < 0.01) and their uses (*Z* = 3.96; p< 0.001): they reported 4.66 (±3.31) species and 15.72 (±11.40) uses, while women 2.32 (±1.43) species and 5.08 (±4.72) uses. There is no consensus in the literature about the effect of gender, though women are generally shown to hold a wider competence concerning medicinal plants than men [66]. Ahmad et al. [48], studied medicinal resources in the mountainous region of Chail valley (Pakistan), observed that females had a higher knowledge about the preparation and administration of herbal drugs compared to males. Similar was observed by [67] in a study concerning the ethnobotanical knowledge of Fulni-ô in north-eastern Brasil, our finding could be explained considering the harvesting dynamics, in which men are the main collectors of medicinal plants growing in the local forest. As for education level, 21 informants (33% of all informants) were uneducated, 8 (12%) received only some primary education 20 (31%) had attended middle or intermediary school and 9 (14%) held a university degree. The education level of informants proved to be significantly associated with both the number of species (*H* = 10.09; *p* < 0.05) and the number of uses (*H* = 38.58; *p* < 0.001). Informants who were less educated (from primary to middle education level) had highest medicinal plant knowledge “Table 3”. It’s important to note that this result is affected by the relationship existing between the education grade and the work activities of the informants: traditional healers having higher level of medicinal knowledge (see below) belonged to the group with lower education level; on the contrary housewives, knowing the lowest number of species and uses, were all uneducated. Informants with higher education were the less knowledgeable: it’s possible to presume that a better scholastic career may expose people to the influence of the academic knowledge and to the allopathic medicinal practices. During the study, seventeen traditional healers having ages ranging from 45 to 60 years were interviewed. Most of the healers were involved in healing practices from more than two to five years and were vastly experienced while only five healers had more than 10 years of experience. When grouped according to profession, Kruskal-Wallis tests show that there is a significant difference among informants in both the number of known medicinal plants and the number of different uses (*H*: 38.51; *p* < 0.001). As we expected, both the mean number of species and the mean number of uses resulted higher for “professional” healers (traditional healers + midwives) *vs* laypeople “Table 3”. Yet, in Rawalakot even the knowledge held by laypeople appears more considerable: lay villagers also reported 42% of the species mentioned by healers. The role of laypeople in preserving and transmitting the ethnomedicinal knowledge has been pointed out by some studies (see for example Bruschi *et al*. [68]), in particular, laypeople were reported to know and use medicinal plants mainly to treat common ailments such as digestive troubles, injuries and wounds, cough, headache. People turn instead to healers for other diseases considered more severe.

**Table 3:** Informants and knowledge about TAB. F = female; M = male.

informants	#		# KNOWN SPECIES		# CITED DIFFERENT USES	
	F	M	F	M	F	M
**Total**	25	39	2.32±1.43	4.66±3.31	5.08±4.72	15.72±11.40
**Age class**						
19–30	9		1.33±0.55		3.00±1.11	
31–40	18		3.38±2.99		10.00±9.93	
41–50	17		4.53±3.02		17.65±3.02	
51–60	20		4.50±3.03		12.55±8.28	
**Education level**						
Uneducated	21		2.29±1.27		6.43±4.43	
Primary school	14		5.50±4.31		14.36±11.51	
Intermediary school	8		3.00±1.52		10.50±9.28	
Middle school	12		5.08±3.14		20.0±14.01	
University degree	9		3.33±2.18		10.88±8.49	
**Profession** Traditional healers and midwives	20		7.15±2.96		25.03±8.00	
Housewives	8		1.87±0.83		5.00±2.98	
Teachers	13		2.38±0.87		6.15±3.16	
Farmers	10		2.10±1.10		5.50±2.81	
Other	13		2.30±1.37		5.92±4.42	

### Comparison with other studies in neighboring regions and Novelty

**Fig 5:**
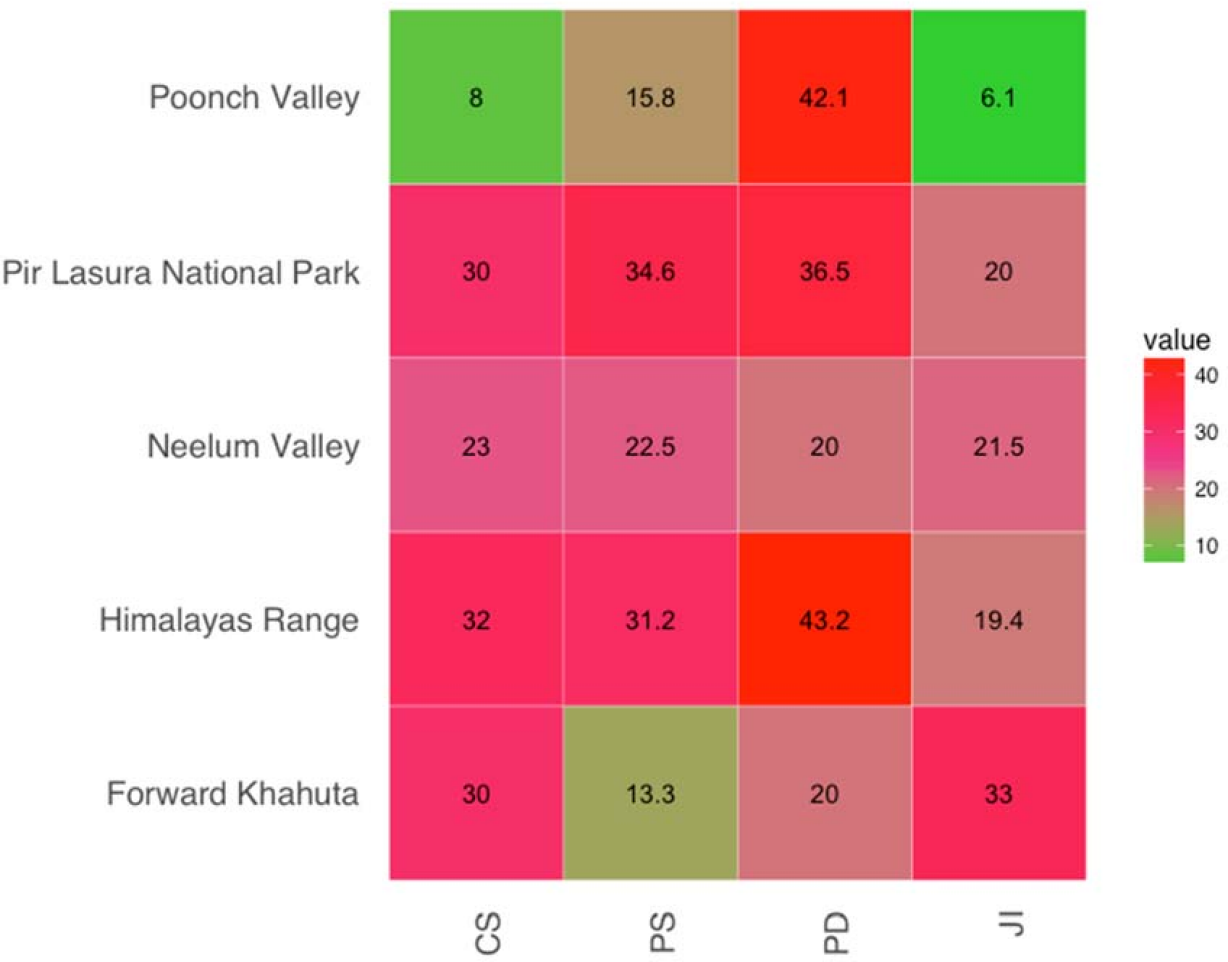
Heat map showing percentage similarity, dissimilarity and Jaccrad Index (JI) of Pearl Valley with neighbouring areas.

When we compared the data from this study with the findings of other studies carried out in neighboring areas, we observed a percentage of similarity in uses of plant species ranging from 13.33% [69] to 34.62% [70] with an average value of 22.53%. The percentage of dissimilarity ranged from 43.20 [71] to 20.00 [72] with an average value of 37.03 “Fig 5”. The maximum level of similarity was found with studies conducted by Ch et al. [69] and Kayani et al. [71], which showed JI values of 32.97 and 19.39, respectively, while the lowest index of similarity was found with the study conducted by Khan et al. [26]which had a JI of 6.11. As observed in other studies [71, 73–75], villagers inhabiting neighboring areas tend to use the same medicinal plants. Presumably, the plant communities occurring in closer areas have more similar plant uses than the communities of more distant areas. A high level of similarity might also be attributed to the fact that the communities living in nearby areas have the same sociocultural values and have more opportunities to exchange their traditional knowledge. The results of this survey provide new insights into the knowledge about medicinal plants of Pakistan: 60% of the plant uses recorded in this research have not been previously reported for AJ&K. Moreover, some of the uses reported by our informants were unknown in the worldwide’s literature “Table 4” Examples include the use of *Opuntia dillenii* for the treatment of joint pain, of *Dalbergia sissoo* for hair growth, and of *Pistacia chinensis* ssp. *integerrima* for wound healing. Species listed in “Table 4” could be appropriate candidates for further studies addressed to the development of new drugs. The number of first time reported plants in the present study indicated a high degree of ethnobotanical novelty for the studied area and confirmed the importance of ethnomedicinal research, also when dealing with already known medicinal plants.

**Table 4:** Novel uses of plant in Pearl Valley Tehsil Rawalakot, District Poonch AJ&K.

**Plants Names**	**Novel Uses**
***Opuntia dillenii* Haw.**	Paste of whole plant used for treating joint pain
***Dalbergia sissoo* Roxb.**	Leaves extract used for hair growth
***Pistacia chinensis* ssp. *integerrima* (J. L. Stewart) Rech.**	Bark paste used as cicatrizant
Pinus roxburgii Roxb.	Resin use to stop bleeding
Adiantum capillus-veneris L.	Leave decoction used against measles
Aesculus indica (Wall.ex Camb.) Hook.f.	Seed powder use against leucorrhoea
Alysicarpus bupleurifolius L. D.C	Leaves fresh juice use as blood purifier
Anaphalis adnata D.C	Leaves powder used as cicatrizant
Bergenia ciliate (Haw.) Sternb.	Root juice and leaves to remove kidney stone
Caltha alba var. alba Camb. var. alba	Aerial parts juice used as sedative
Poa nepalensis Walls ex. Duthie.	Leaves decoction used against body and head lices

The annual temperature in Pearl Valley was 38°C during summer and– 3°C during the winter and rainfall ranged from 500 to 2000 mm. Due to huge variation in climatic conditions the region is biodiversity hotspot and contains representative of almost all life forms. The study areas selected for comparision with present study had low temperature during in winter and moderate temperature during summer. Geographic distance, climatic conditions and cultural values of Pir Lasura National Park are similar to study area so there are more chances of exchange of knowledge between these areas. The argument was supported by highest value of similarity index 34.62% between two areas. Similarly, the areas that are far apart from study area had least similarity in uses, thus plant the present in these areas with similar uses and methods of preprations to those of plants in study area, should be considered to have higher potential than other plants that may be used for a particular disease in only one culture.

### Conservation status of the plants

Projects aimed to safeguard and enhance ethnobotanical knowledge about medicinal plants must be associated with to specific strategies for the conservation and sustainable use of plants in order to ensure the convergence of development and conservation goals [76]. Extractive activity can play an important role in local health care systems, but a high collection rate of medicinal plants can lead to over-exploitation and can have a strong impact on local plant diversity. According to the IUCN Red List (version 3.1), *Pinus roxburghii* and *Zanthoxylum armatum* were reported as vulnerable (VU), while *Punica granatum* was indicated as endangered (EN); people living in the area use different parts of these plants not only as medicine but also for more destructive uses such as fuelwood, construction, and furniture. Several other plants shown in Table 2 are reported to be of least concern (LC). The most important factors considered by local informants as threats to the survival of the flora were deforestation, agricultural expansion, overgrazing, fire, and drought. The continuous environmental degradation of the collection habitats could hasten the depletion of medicinal plants and the rarefaction of the associated knowledge. In recent years, some traditional practitioners have started to cultivate medicinal plants in their home gardens, but this effort seems to be insufficient given that only 18% of the medicinal plants reported in this study were cultivated.

## Conclusions

This study was the first to document the uses of medicinal plants in the District Rawalakot area. Our data show that medicinal plants are an important source for local people (136 species reported by 64 informants) and point out that both traditional healers and laypeople have developed a rich knowledge base on their experience. In more remote areas, where health services have struggled to reach, this knowledge can provide first aid for treating the most common diseases occurring in the area. The plants having high values of UV and RFC are biologically active and had good healing potential for specific ailments. A study area was located at higher altitude so respiratory problems were most common diseases in the area, similarly due to low temperature during most of the days of year skin problem were ranked second among different cited diseases. A significant effect of gender and age on the distribution of ethnobotanical knowledge was recorded during an interview and was confirmed statistically. Spearman’s correlation analysis depected that there is positive differences between the age and the number of both number of mentioned species (rs = 0.49; *p* < 0.05) and different uses (rs = 0.45; *p* < 0.05), indicating that there is a trend of older people being more knowledgeable than younger people. When we compare the knowledge held by men and women, men showed a much higher knowledge on medicinal plants (*Z* = 3.20; *p* < 0.01) than women. The distance between areas was the main determinant of change in jaccard index value. Areas located closer to each other have more opportunities for exchange of traditional knowledge and similarities among neighboring areas also depend upon environmental factors. The highest similarity between the study area and Pir Lasura National Park may be due to the sharing of a similar flora and the cross-cultural exchange of medicinal plant knowledge in past and present. Less similarity between the areas may be because they are so distantly related that they are very unlikely to have communicated medicinal plant uses to each other, they are disconnected through mountain ranges and other cultural variations. Any related plants used by these areas to treat related diseases are independent discoveries. We believe that results of the present study may represent useful information that could contribute to preserving the local indigenous knowledge about the use of medicinal plants and also attract the future generations toward the traditional healing practices. Moreover, this study provides baseline information for further studies aimed at the identification and isolation of bioactive molecules that can serve as starting materials in the discovery of new plant based drugs and also create awareness about the conservation and protection of biocultural diversity.

## Supporting information

S1 File Sample of Questionnaire used during field survey for obtaining ethnobotanical information. (DOCX)

S2 File Table of Jaccard’s Index (JI) of pearl valley Tehsil Rawalpindi, District Poonch AJ&K. (DOCX)
