# Supplementary material for: Exploration of ethno-medicinal knowledge among rural communities of Pearl Valley; Rawalakot, District Poonch Azad Jammu and Kashmir
Source: PLoS One. 2017 Sep 8;12(9):e0183956. doi: 10.1371/journal.pone.0183956 (PMC5590857; doi:10.1371/journal.pone.0183956)
Supplement: S1 File — (DOCX) [file pone.0183956.s001.docx]

**(ANNEXURE-1)**

**PROFORMA FOR THE INTERVIEW FROM THE INHABITANTS REGARDING ETHNOBOTANICAL USES OF PLANT SPECIES**

**Date…………**  **Area/ Locality**:………….

**Name/ Address of Respondent: _____________________________________________**

**Enumerator:**

**Botanical Name: Vernacular Name:**

**Language:** **Locality:**

**USES**

**A-Medicine:**

 Part of plant used: ____________________________________

Method of preparation: ____________________________________

Type of Ailment: ____________________________________

Recipe __________________________________

**B-Veterinary:**

 Disease: ____________________________________

Part of plant used: ____________________________________

Method of preparation: ____________________________________

**C-Fodder:**

All parts of Plants: **[ ]**

Leaves: **[ ]**

Fruits: **[ ]**

Seeds: **[ ]**

Availability of forage: **[ ]**

**D-Fuel:**  **[ ]**

**E-Any other usage/Cultural Beliefs:**

**Informant information**

Age: Education:

Gender: Lay man or traditional health practitioners

If traditional health practitioners then experience:

**(ANNEXURE-2)**

**Date: ……………..**

**INFORMATION OF RESPONDENT**

1. Name………………………………………………………………………………

2. Age

(a) 15-35 (b) 36-50 (c) 51-75 (d) 76-100

3. Sex

(a) Male (b) Female

4. Education:

(i) Primary (ii) Middle (iii) Secondary (iv) Higher secondary (v) Illiterate

5. Occupation:

(i) Govt. Job (ii) Private Job (iii) Agriculture (iv) Other………………

6. Household composition:

(i) Men……………… (ii) Women………………. (iv) Children…………..

(iii) Total members……………………

7. Sources of income

(i) Job (ii) agriculture (iii) other……………………..

8. Land owner: (a) yes (b) no

9. Land category

(a) Rented in …………

(b) Rented out ……….

(c) Shared …………..

**Information of plants used as FODDER:**

10. Do you have livestock?

(a) yes (b) no

11. If yes, then which one of the followings?

(a) Cows (b) buffaloes (c) goats (d) sheep (e) donkeys

(f) Others……………………………….

12. What is the number of livestock?

(a) Cows = (b) buffaloes = (c) goats = (d) sheep = (e) donkeys=

(f) Others =

13. How you feed your livestock?

(a) Rangeland (b) crop stalks (c) wheat straw

(d) Other……………………………

14. What is the source of fodder/forage?

(a) forest (b) cultivated land (weeds) (c) purchased from market

| Name of Species | Wild/  Cultivated  (W/C) | Tree/Herb/  Shrub/  Grasses  (T/H/S/G) | Part used | Animal preference  (S/G/C/D) | Palatability  (Very high/  High/  Moderate/Less /Non palatable) |
| --- | --- | --- | --- | --- | --- |

| Leaves | branches | Whole  plant | other |
| --- | --- | --- | --- |

**Information of Plants used as FEULWOOD:**

15. Which material do you use for cooking/heating purpose?

(a) gas

(b) wood

(c) kerosene oil

(d) other……………

16. From where you get fuel wood plants?

(a) from forest (b) cultivated land (c) road side (d) marginal areas

(e) other……………

17. Who collect the plants?

(a) men (b) women (c) childern

18. Which plants are used as fuel wood, name and part used.

| Name of Species | Wild/  Cultivated  W/C | Tree/Herb/  Shrub  T/H/S | Part used | Preference rank | Reasons |
| --- | --- | --- | --- | --- | --- |

| Whole  plant | Branches | Stem | Other |
| --- | --- | --- | --- |

19. Which form of wood is used?

(a) fresh (b) dead (c) dry

(d) other

**Information of Plants used as TIMBER:**

20. Did the plants are used for timber purpose?

(a) yes (b) no

21. From where you get these plants?

(a) forest (b) cultivated land (c) market

22. Name and part of plant used for timber.

| Name of Species | Wild/  Cultivated  W/C | Abundance  Rare/  Common | Plant use | Preference rank | Reasons |
| --- | --- | --- | --- | --- | --- |

| ceiling | window | door | Others |
| --- | --- | --- | --- |

**Information of Plants used as MEDICINES**:

**i. Ethno veterinary Use:**

23. Which diseases of livestock are common in the area?

…………………………………………………………………………………………

24. What is the sign/symptom of this disease or how you identify this disease?

………………………………………………………………………………………………

25. Which kind of treatment you gave to your livestock?

(a) western medicine (b) herbal

26. If herbal, then reason for this use

(a) most effective (b) easily available (c) free of cost

(d) other……………..

27. Name and part of plant used for medicines.

| Name of Species | Disease | Tree/ Herb/  Shrub | Part used | Recipe |
| --- | --- | --- | --- | --- |

| Leaves | Fruit | Flower | Root | Whole  plant |
| --- | --- | --- | --- | --- |

|  |  |  |  |  |  |  |  |  |
| --- | --- | --- | --- | --- | --- | --- | --- | --- |
|  |  |  |  |  |  |  |  |  |
|  |  |  |  |  |  |  |  |  |
|  |  |  |  |  |  |  |  |  |
|  |  |  |  |  |  |  |  |  |
|  |  |  |  |  |  |  |  |  |

28. Which species are becoming rare and why?

(i) …………………………………………………………………………

(ii)…………………………………………………………………………

(iii) ………………………………………………………………………..

(iv) ………………………………………………………………………..

(v) …………………………………………………………………………

**ii. Ethno medicinal Use:**

29. Which diseases of human are common in the area?

………………………………………………………………………………………

30. What kind of treatment you get?

(a) Allopathic (b) Herbal

31. If herbal, than what is the reason?

(a) easily available (b) most effective (c) free of cost

(d) other………….

32. From where you get these plants?

(a) from forest (b) from own land (c) Weeds

33. Name and part of plant used for medicines.

| Name of Species | Disease | Tree/ Herb/ Shrub | Part used | Recipe |  |  |  |  |
| --- | --- | --- | --- | --- | --- | --- | --- | --- |

| Leaves | Fruit | Flower | Root | Whole  plant |
| --- | --- | --- | --- | --- |

34. Which species are becoming rare and why?

(i) …………………………………………………………………………

(ii)…………………………………………………………………………

(iii) ………………………………………………………………………..

(iv)………………………………………………………………………..

(v) …………………………………………………………………………
