# Supplementary material for: Exploration of ethno-medicinal knowledge among rural communities of Pearl Valley; Rawalakot, District Poonch Azad Jammu and Kashmir
Source: PLoS One. 2017 Sep 8;12(9):e0183956. doi: 10.1371/journal.pone.0183956 (PMC5590857; doi:10.1371/journal.pone.0183956)
Supplement: S2 File — (DOCX) [file pone.0183956.s002.docx]

Table 4: Jaccard's Index (JI) of Pearl valley Tehsil Rawalakot, District Poonch AJ&K

| Area | Study year | Number of study participants | Number of recorded plant species | Species cited per informant | Total species common in both area | Species enlisted only in aligned areas | Species enlisted only in study area | % of plant with similar uses | % of dissimilar uses | JI | Citation |
| --- | --- | --- | --- | --- | --- | --- | --- | --- | --- | --- | --- |
| Himalaya range | 2015 | 29 | 125 | 4.31 | 32 | 93 | 104 | 31.20 | 43.20 | 19.39 | Kayani et al., 2015 |
| Neelum valley | 2011 | 35 | 40 | 1.14 | 23 | 17 | 113 | 22.50 | 20.00 | 21.50 | Mahmood et al., 2011 |
| Pir Lasura National Park | 2015 | 70 | 104 | 1.49 | 30 | 74 | 106 | 34.62 | 36.54 | 20.00 | Amjad et al., 2015 |
| Forward Khahuta | 2013 | 40 | 45 | 1.13 | 30 | 15 | 106 | 13.33 | 20.00 | 32.97 | Ch et al., 2013 |
| Poonch Valley | 2012 | 37 | 19 | 0.51 | 8 | 11 | 128 | 15.79 | 42.11 | 6.11 | Khan et al., 2012 |
| Average |  | 41.83 | 83.67 | 2.13 | 26.67 | 57.00 | 109.33 | 22.53 | 37.03 | 19.84 |  |
